# Supplementary material for: Association analysis of Mycoplasma pneumoniae 23S rRNA gene mutation with refractory Mycoplasma pneumoniae pneumonia in children
Source: PLoS One. 2026 Jan 29;21(1):e0341580. doi: 10.1371/journal.pone.0341580 (PMC12854454; doi:10.1371/journal.pone.0341580)
Supplement: Attachment — Submitted filename: review comments.pdf [file pone.0341580.s001.pdf]

The manuscript titled "Correlation analysis of *Mycoplasma pneumoniae* 23S rRNA gene mutation with refractory *Mycoplasma pneumoniae* pneumonia in children" is a clinical relevant retrospective study written in a well organized manner. It explores the relationship between mutations in 23S rRNA of *Mycoplasma pneumoniae* and refractory *Mycoplasma pneumoniae* pneumonia (RMPP) disease in children. Study makes significant impact in the given rising prevalence of macrolide-resistant *M. pneumoniae* in Asia, and the authors provide a comprehensive data analysis from 205 pediatric cases.

However, while the study demonstrates statistical correlations and potential clinical implications, several **methodological, interpretive, and linguistic issues** should be addressed before publication.

Major Comments

1. Scientific Content and Study Design

Study includes adequate sample size (n-205) for a single cluster analysis with clear inclusion and exclusion criteria. Using logistic regression and ROC analysis strengthens the interpretation on clinical relevance which focuses on predictors of RMPP progression. With these strengths I recommend to emphasize the limitation of retrospective design in the discussion (also in abstract) and also to discuss potential confounding factors like heterogeneity or selection bias.

RMPP definition: it is not clear "persistent fever ≥ 7 days" means fever duration after initiation of macrolide therapy or total fever duration.

Mutational analysis: Mentions "PCR with TaqMan fluorescent probe," however, there is no details on primers, probe sequence, QC or mutation confirmation rate. This information is necessary for reproducibility by other labs.

Statistical clarity:

The logistic regression model variables should be listed (what are the variable). Confidence intervals in Table 8 are wide (OR 17.8, Cl 2.9-107.9) which indicates instability please address multicollinearity or small sample bias.

While concluding I feel authors overstate "drug-resistant mutation lead to RMPP" and given the retrospective nature of the study "association of mutation" might sound better.

Language and Style

Overall Impression:

The manuscript is written in formal scientific English but would benefit from editing for clarity, grammar, and concision.

Specific Issues:

1. **Repetition:**
   Some phrases and results are repeated in both the abstract and results section (e.g., "D-dimer emerged as the most predictive risk factor") make it short and simple.

2. **Tense Consistency:**
   Use **past tense** for methods and results ("was conducted," "showed") and **present tense** for interpretations.

3. **Terminology:**

   1. Replace "drug-resistant group" with "macrolide-resistant group" for precision.
   2. Avoid "datas" (plural error in Methods 2.1) → "data were accessed."
   3. Avoid unnecessary capitalization (e.g., "Paediatrics Department," "Bronchoscopic Interventional Alveolar Lavage").

4. **Sentence Structure:**
   Some sentences are long and complex. Break them into shorter, clearer statements. Example:
   "MP infects the respiratory tract, causing direct damage to the respiratory epithelium and lungs via adherence and the secretion of toxic substances, such as those implicated in community-acquired respiratory distress syndrome (CARDS)."
   can be simplified as
   "*M. pneumoniae* damages respiratory epithelium through adhesion and toxin secretion, including CARDS toxin."

5. **Abstract:**
   Limit to 300 words. Current abstract slightly exceeds and reads as overly detailed; consider shortening the methods description.

6. **Figure and Table Titles:**
   Ensure all tables/figures are self-explanatory and units are standardized (ng/mL, days). Avoid abbreviations in headings (e.g., “PE,” “PB”) unless defined.

7. **References:**
   Many references are outdated (some >10 years). Include more recent studies (2022–2024) on macrolide-resistant *M. pneumoniae* in children, especially from China.

Data Presentation

- Tables are comprehensive but dense. Consider moving some comparisons (e.g., mixed infection data) to supplementary material.

- ROC figure is appropriate; ensure AUC values are reported with 95% CI.

Recommendation

Major Revision

The study addresses an important and under-reported topic with robust data and sound analysis. However, the following are needed before *PLOS ONE* publication:

1. Clarify methodology and statistical modeling.

2. Refine language and streamline redundant sections.

3. Ensure compliance with *PLOS ONE* data-sharing and formatting guidelines.

4. Tone down causal claims and strengthen limitations discussion.
