# Supplementary material for: Association analysis of Mycoplasma pneumoniae 23S rRNA gene mutation with refractory Mycoplasma pneumoniae pneumonia in children
Source: PLoS One. 2026 Jan 29;21(1):e0341580. doi: 10.1371/journal.pone.0341580 (PMC12854454; doi:10.1371/journal.pone.0341580)
Supplement: Attachment — Submitted filename: Response to Reviewers.docx [file pone.0341580.s003.docx]

**Response to Reviewer**

Dear Editors and Reviewers,

Thank you for the opportunity to revise and resubmit our manuscript. We sincerely appreciate the time and effort the reviewers have dedicated to providing insightful and constructive comments on our work. These comments have been invaluable in helping us strengthen the scientific rigor, clarity, and overall quality of the manuscript.

We have carefully considered each point raised and have made comprehensive revisions to address all concerns. Below, we provide a point-by-point response to the reviewers’ comments, detailing the changes made. All modifications in the revised manuscript are highlighted using the ‘Track Changes’ function in Microsoft Word.

We believe the revised manuscript has been significantly improved and hope it now meets the high standards required for publication.

Sincerely,

Aimin Li
On behalf of all co-authors

**Reviewer #1**

We are grateful to you for the overall positive assessment and the constructive feedback provided in the attached comments. We have meticulously addressed all the methodological, interpretive, and linguistic points raised in the separate file. Our detailed point-by-point responses are integrated below, and all corresponding revisions have been made in the manuscript. We believe these changes have significantly strengthened the study’s clarity and robustness.

**Reviewer #2**

We thank you for highlighting the need for more methodological detail. In response, we have **substantially expanded Section 2.5 “Experimental Methods and Results Analysis”**. Specifically, we have added:

1. A detailed subsection (**2.5.1 Experimental Methods**) describing the commercial kit (Jiangsu Molabio), its principle (TaqMan fluorescent PCR), target genes (P1 gene and 23S rRNA domain V), and the interpretation of results based on Ct values.
2. Citations for the kit and equipment used.
3. Brief descriptions and references for other key assays mentioned by the reviewer, including the *MP*-IgM/Ab titer detection method (passive agglutination, Kit: Serodia-Mico II) and the multiplex PCR for respiratory pathogens (Kit: Daan Gene). These additions are now in Sections 2.3 and 2.5.

**Reviewer #3**

1. The term correlation is not appropriate for the analysis of the categorical variables. It should be restricted to analysis of the continuous variables. The term association will be better here, especially in the title.

**RE:** We agree with the reviewer’s suggestion. The title has been changed to: **“Association analysis of Mycoplasma pneumoniae 23S rRNA gene mutation with refractory Mycoplasma pneumoniae pneumonia in children”**. This more accurately describes the analysis involving categorical variables.

2. The methodology does not describe at least in brief, with citation of the methodology, how the various laborartory tests were done.

a. How was the MP-DNA done ?
b. How were the aforementioned mutations detected? Was sequencing done?
c. How were the MP-IgM and Ab titers detected and by which kit or methods ?
d. Nucleic acids for seven respiratory pathogens – how was it tested, which samples and which kits?
e. The different laboratory tests like wbc, crp , ldh, d-dimer and esr and PCT were included at what time point after admission? Is it the first test soon after admission?

**RE:** We appreciate the reviewer’s request for greater methodological transparency. We have comprehensively revised the **Materials and Methods section (Section 2)** to address all these points:

**2a & 2b: MP-DNA and Mutation Detection:** Detailed procedures for nucleic acid extraction, PCR setup, cycling conditions, and result interpretation for detecting *MP*-DNA and the A2063G/A2064G mutations are now provided in **Section 2.5.1**. We specify the use of a commercial fluorescent PCR kit, which is the standard and efficient method for screening these known point mutations in our clinical setting, as sequencing is not routinely performed for this purpose.

**2c: MP-IgM and Ab Titers:** MP-IgM and antibody titer detection employ the passive agglutination method（PA）, which is the primary laboratory technique for measuring serum MP-IgM antibodies. A single serum antibody titer ≥1:160 serves as the criterion for recent MP infection. The kit utilizes the “Mycoplasma pneumoniae Antibody Detection Kit (Passive Agglutination Method), Serodia-Mico II,” manufactured by Zhuhai Lizu Reagent Co., Ltd.

**2d: Seven Respiratory Pathogens:** The nucleic acids of seven respiratory pathogens (rhinovirus, respiratory syncytial virus, adenovirus, influenza A virus, influenza B virus, human parainfluenza virus, and Mycoplasma pneumoniae) were detected using a multiplex fluorescent PCR method with the “Respiratory Pathogen Multiplex Detection Kit” from Daan Gene. The manufacturer of this kit is Sun Yat-sen University Daan Gene Co., Ltd.

**2e: Timing of Laboratory Tests:** We have clarified in **Section 2.3** that the laboratory parameters (WBC, CRP, LDH, D-dimer, ESR, PCT) reported and analyzed in this study were collected from the **first blood draw upon hospital admission**.

3. How were the variables selected for the multivariable regression analyses?

**RE:** Thank you for this question. The variables for the multivariable logistic regression model were selected in a two-step process:

**Univariate Screening:** All clinical, laboratory, and imaging variables were first compared between the RMPP and non-RMPP groups using appropriate univariate tests (chi-square, t-test, Mann-Whitney U). Variables with a **P-value < 0.05** in these univariate comparisons were considered potential candidates.

**Clinical and Statistical Refinement:** To avoid overfitting the model given our sample size, we then selected the most clinically relevant and statistically robust predictors from the candidate pool. This process led to the final inclusion of **macrolide-resistant gene mutation, duration of fever, and D-dimer level** in the model, as detailed in **Section 3.8**. This selection rationale has also been clarified in the revised manuscript.

4. None of the continuous variables in the table 1 are expressed as medians with IQR. Were all the subsets of the continuous variables for RMPP and non RMPP really having normal distribution? It seems highly unlikely. This is also reflected in the variables like the durations and the PCT (eg 0.32 vs 0.23, p – 0.026) values among the 2 groups look very similar, but have significant p value. Kindly recheck the normality of distribution of the continuous variables for each subset of drug resistant and non drug resistant groups and perform the t test only for those where the give variable has normal distribution for each of drug resistant and non drug resistant groups. If the variables are non normal in any of the 2 subgroups, alternative tests of significance needs to be used.

**RE:** We sincerely thank the reviewer for this critical methodological point. You are absolutely correct to question the assumption of normality for all continuous variables. Upon re-examination, we found that several key variables (including durations of fever, treatment, and laboratory markers such as PCT) did not follow a normal distribution within the compared subgroups.

In response to your suggestion, we have **completely re-analyzed all continuous data** in the study as follows:

1. **Normality Assessment:** Each continuous variable was tested for normality within the macrolide-resistant and non-resistant groups (and separately within the RMPP and non-RMPP groups for Table 2) using the Shapiro-Wilk test.
2. **Revised Descriptive Statistics:** Variables with a non-normal distribution are now **appropriately presented as median (interquartile range, IQR)**, M (P25, P75).
3. **Revised Inferential Statistics:** For between-group comparisons of non-normally distributed variables, we have applied the **Mann-Whitney U test (Wilcoxon rank-sum test)** instead of the independent samples t-test.

**Key Outcome of Re-analysis:**
The re-analysis confirmed your observation. Notably, the difference in **PCT levels between the macrolide-resistant and non-resistant groups is no longer statistically significant** (*P* > 0.05). This correction has been made in the revised **Table 1**. Tables 2 has been similarly reviewed and corrected where necessary to ensure all statistical tests are appropriate for the data distribution.

We appreciate this suggestion, which has significantly improved the statistical rigor of our manuscript. The revised tables and the updated statistical description in the **"2.6 Statistical methods"** section reflect these changes.

5. The table 2 also needs to be checked similarly for normality of distribution of the continuous variables.

**RE:** Thank you for this important reminder. We have systematically re-evaluated all continuous variables presented in Table 2 following the same rigorous process applied to Table 1.

1. **Normality Assessment**: The distribution of each continuous variable within both the RMPP and non-RMPP subgroups was tested using the Shapiro-Wilk test.
2. **Revised Analysis**: For variables that did not follow a normal distribution in either subgroup, between-group comparisons were performed using the **Mann-Whitney U test** instead of the t-test, and data are now presented as **median (IQR).**
3. **Updated Results**: The revised statistical analysis confirms the significant differences (*P* < 0.001) originally reported for key variables (e.g., duration of fever, CRP, LDH, D-dimer, ESR, PCT, and length of hospitalization) between the RMPP and non-RMPP groups. **The conclusions regarding these risk factors remain unchanged and are now supported by more appropriate non-parametric tests where needed.**

**The corrected Table 2 has been updated in the manuscript accordingly.**

6. Are the antibiotic/hormonal therapy groups in the table 7 mutually exclusive?

**RE:** Thank you for your critical question. The groups in Table 7 are mutually exclusive based on the actual final treatment regimen received by each patient. They are categorized as follows for statistical comparison: 1) Novel tetracycline/levofloxacin**+** methylprednisolone; 2) Azithromycin **+**methylprednisolone; 3) Azithromycin alone; 4) Novel tetracycline/levofloxacin alone. As noted in the discussion (Section 4), the choice of regimen reflects real clinical decisions influenced by disease severity and suspected resistance.

a. Why were the antibiotics other than macrolides used in the non-RMPP groups?

**RE:** This is a very important observation that highlights a key aspect of our clinical context. As you noted, 86 (67.2%) of the 128 non-RMPP patients harbored macrolide-resistant mutations. In clinical practice during the high-resistance epidemic period of 2023-2024, physicians often initiated alternative antibiotics (novel tetracyclines or levofloxacin) early in patients presenting with more severe initial symptoms or signs suggestive of potential treatment failure, even before completing a full 7-day course of macrolides. This proactive management likely prevented some patients from progressing to meet the formal RMPP criteria, leading to their inclusion in the non-RMPP group.

b. Does it not interfere with the grouping in the study, where the RMPP group is defined when the patient worsens despite receiving 7 days of macrolide?

**RE:** You have identified a significant methodological consideration. Yes, this clinical practice introduces a potential **confounding factor**. By definition, the RMPP group consists of patients who failed a ≥7-day macrolide course. The non-RMPP group, however, is **not a pure “macrolide-responsive” group but rather a “clinically favorable outcome” group**. This group’s favorable outcome may be partly attributable to the early switch to more effective antibiotics for those with resistant strains, rather than solely to macrolide efficacy. This could attenuate the observed association between macrolide-resistant mutations and the outcome of “clinical macrolide treatment failure” (i.e., RMPP).

c. By this, is it not implicated that non-RMPP group will be one that recovers after < 7 days treatment with only macrolides?

**RE:** This would be the ideal scenario for a controlled study design. However, in this real clinical practice, retrospective cohort, the non-RMPP group is defined by not meeting the RMPP criteria, which includes both: 1) patients who responded well to macrolides within 7 days, and 2) patients who were switched early to non-macrolide antibiotics and subsequently improved, thus never entering the “7-day macrolide failure” observation window. Therefore, the non-RMPP group is heterogeneous in terms of initial treatment response. We acknowledge this as a **limitation** of the retrospective design. In the revised manuscript, we will add a paragraph to the Discussion/Limitations section to explicitly address this issue and its potential impact on the interpretation of the association between genotypic resistance and the clinical outcome of RMPP.

7. Table 8:
a. How were the significant factors selected for the multiple logistic regression analysis.

**RE:** Thank you for your valuable comments. Regarding your questions about the variable selection method and criteria for the multivariate logistic regression analysis, we provide the following detailed explanation:

Based on univariate analyses (including chi-square test, t-test, and Mann–Whitney U test), we first identified all clinical and laboratory indicators that showed statistically significant differences (*P* < 0.05) between the RMPP and non-RMPP groups. These variables included: duration of fever, time to defervescence after azithromycin treatment, length of hospital stay, CRP, LDH, D-dimer, ESR, procalcitonin PCT, presence of pulmonary consolidation, pleural effusion, need for bronchoscopic alveolar lavage, and occurrence of extrapulmonary complications. These factors, demonstrating significant association with RMPP in initial comparisons, were considered candidates for the multivariate analysis.

b. What criteria were used? Was univariate regression analysis performed to select important variables for the multivariate analysis?
**RE:** Yes, we performed univariate logistic regression analyses for each of the variables identified as significant in the initial group comparisons. Variables with *P* < 0.05 in the univariate logistic regression were retained for further consideration. To build a parsimonious and clinically meaningful multivariate model, we then applied the following selection criteria:

(1) Statistical significance in univariate logistic regression (*P* < 0.05); (2) Clinical relevance and biological plausibility based on existing literature (e.g., fever duration and D-dimer are established markers of disease severity and complication risk in pneumonia); (3) Assessment of multicollinearity among candidate variables to ensure model stability.

Following these criteria, the variables **Drug-resistance mutation, duration of fever, and D-dimer** were entered into the final multivariate logistic regression model. This model confirmed these three factors as independent risk factors for RMPP (*P* < 0.05), as presented in Table 8.

8. As mentioned in the results, 67.2% of the non RMPP had the drug resistant mutation.
a. How is this justified?
b. Does this not indicate that the detection of mutation is not a reliable test for detection of macrolide resistance?
c. Or is this because the groups used in the study are not proper.
d. Especially the non RMPP group, most probably includes patients that were not treated initially only by macrolides as evident in table 7.
e. If this is the case, the study is comparing the cases that did not respond to macrolides for seven days to a very heterogeneous group which received a variety of different initial antibiotics other than macrolides and hence the discrepancy in the mutation rates in the 2 groups. This not a good study design and the conclusions of the statististical analysis, hence, may not be reliable. The groups of RMPP and non-RMPP may need to be reviewed, which will affect the whole analysis and conclusions throughout the manuscript.

**RE:** We sincerely thank the reviewer for raising these critical and insightful points, which touch upon the core interpretation of our findings. We address each sub-question below:

a. Reason for high mutation rate in the non-RMPP group (67.2%):
This observation is indeed important and does not necessarily invalidate our approach. A high prevalence of macrolide-resistant *M. pneumoniae* (MRMP) in the general MPP population has been widely reported, especially during the study period (2023-2024), which was a peak epidemic season with reported resistance rates exceeding 90% in some regions [references 28-30]. **Therefore, a substantial proportion of patients in the non-RMPP group likely harbored MRMP strains but did not progress to refractory disease. This suggests that the presence of the A2063G/A2064G mutation is a necessary but not sufficient condition for developing RMPP. Other host (e.g., immune response) and pathogen (e.g., bacterial load, virulence factors) factors likely determine whether an MRMP infection becomes refractory.**

References:

28.	Chen Y-C, Hsu W-Y, Chang T-H. Macrolide-Resistant Mycoplasma pneumoniae Infections in Pediatric Community-Acquired Pneumonia. *Emerg Infect Dis.* 2020;26(7):1382-1391.

29.	Kim K, Jung S, Kim M, Park S, Yang H-J, Lee E. Global Trends in the Proportion of Macrolide-Resistant Mycoplasma pneumoniae Infections: A Systematic Review and Meta-analysis. *JAMA Netw Open.* 2022;5(7):e2220949.

30.	Wang X, Li M, Luo M, et al. Mycoplasma pneumoniae triggers pneumonia epidemic in autumn and winter in Beijing: a multicentre, population-based epidemiological study between 2015 and 2020. *Emerg Microbes Infect.* 2022;11(1):1508-1517.

b. Reliability of mutation detection for macrolide resistance:
The detection of 23S rRNA domain V mutations (A2063G/A2064G) is the **well-established and gold-standard molecular method** for predicting macrolide resistance in *M. pneumoniae*, with a strong correlation to phenotypic resistance in vitro. Our finding of a high mutation rate aligns with the known epidemiology. The discrepancy between genotype (mutation present) and clinical outcome (non-RMPP) reflects the **complexity of clinical disease rather than a failure of the detection method**. The mutation reliably predicts the potential for macrolide treatment failure, but the actual clinical course is modulated by other variables.

c. & d. & e. Appropriateness of grouping and the critical issue of heterogeneous initial treatment:
We fully acknowledge the reviewer's central and most valuable critique regarding the heterogeneity of initial antibiotic therapy in the non-RMPP group, as correctly observed in Table 7.

1. Defense of RMPP/Non-RMPP Grouping: The RMPP definition (persistent fever/clinical deterioration after ≥7 days of macrolide therapy) is **the standard clinical definition adopted by national guidelines**. Its purpose is to identify a clinically distinct, severe phenotype. Our use of this grouping is intended to reflect real clinical practice and outcomes, **not to create a pure “macrolide exposure vs. non-exposure” experimental design.**
2. **Admission of the Confounding Factor**: **The reviewer is absolutely correct.** The non-RMPP group includes patients who received various initial regimens (e.g., tetracyclines/fluoroquinolones alone or in combination, as per Table 7). **Therefore, for a subset of the non-RMPP group, the macrolide therapy component of the RMPP definition was not fully tested**. This is a **limitation** of our retrospective, observational design. The comparison is indeed between **a group that failed macrolide therapy (RMPP) and a group with heterogeneous initial therapy, some of whom might have also failed macrolides had they been used initially.**
3. **Impact on Analysis and Mitigation**: This therapeutic heterogeneity could attenuate the observed association between the mutation and the RMPP outcome. If we could isolate only the patients in the non-RMPP group who received an adequate trial of macrolides, the difference in mutation rates between groups might be even more pronounced.

**In summary**, we agree that the heterogeneity in initial treatment within the non-RMPP group is a significant confounding factor that complicates the direct interpretation of the mutation rate difference. We have incorporated a detailed discussion of this key limitation, its potential impact on our results, and the rationale for our clinical grouping in the revised manuscript (**please see the ‘Limitations’ section in the Discussion)**. We believe that despite this inherent limitation of the real clinical data, our study provides valuable insights into the association between a well-defined severe clinical phenotype (RMPP) and genotypic resistance, while controlling for other strong predictors like D-dimer and fever duration.

**Responses to Reviewer Comments**

We thank the reviewer for the positive assessment of our study’s clinical relevance and organization, and for the detailed suggestions to enhance its methodological clarity and presentation.

All changes made in response to the reviewers' comments are **highlighted in red within the manuscript.**

**Major Comments**

**1. Scientific Content and Study Design**

**Comment 1.1:** “RMPP definition: it is not clear ‘persistent fever ≥ 7 days’ means fever duration after initiation of macrolide therapy or total fever duration.”

**Response:** We thank the reviewer for highlighting this ambiguity. The RMPP definition refers to persistent fever **after the initiation of macrolide therapy**. We have clarified this in the Methods section to ensure precise interpretation.

**Change made:** In **Section 2.2 (Inclusion and exclusion criteria)**, we have revised the RMPP diagnostic criteria to: “…children with MPP **treated with macrolide antibiotics for ≥ 7 days**, but who still exhibit persistent fever, worsening clinical symptoms and lung imaging…” (Page 3, marked in red).

**Comment 1.2:** “Mutational analysis: Mentions ‘PCR with TaqMan fluorescent probe,’ however, there is no details on primers, probe sequence, QC or mutation confirmation rate. This information is necessary for reproducibility by other labs.”

**Response:** We agree that providing methodological details is crucial for reproducibility. The detection was performed using a standardized commercial kit. We have now expanded the description in the Methods section to include the design principle of the primers/probes, the target genes, and the quality control measures incorporated in each run.

**Change made:** We have added a new subsection **(2.5 Experimental Methods and Results Analysis)**. This subsection now includes the following information (Page 4-5, marked in red):

Clarification that primers and TaqMan probes targeted the P1 gene (VIC-labeled) and the A2063G/A2064G mutation sites in the 23S rRNA gene (FAM-labeled).

A statement that the specific primer and probe sequences are proprietary to the commercial kit (Jiangsu Molabio Biotechnology Co., Ltd., China) but the assay principle is as described.

Description of the internal control and the inclusion of no-template controls (NTCs) and kit-provided positive controls (weak and strong) in each run for quality assurance.

Reference to the results analysis table (now Table 1 in Section 2.5.2) which details the Ct value-based interpretation criteria.

**Comment 1.3:** “Statistical clarity: The logistic regression model variables should be listed (what are the variable). Confidence intervals in Table 8 are wide (OR 17.8, CI 2.9-107.9) which indicates instability please address multicollinearity or small sample bias.”

**Response:** We thank the reviewer for this important statistical insight.

**Variable Selection:** We have added a clear description of the variable selection process for the multivariate logistic regression model in the **Results section (3.8)**. We explain that candidates were chosen from variables showing significant univariate differences (P < 0.05), and the final model focused on the most clinically relevant and non-collinear predictors.

**Wide Confidence Intervals:** We acknowledge that the wide confidence interval for the macrolide-resistant gene mutation is a limitation, likely due to the small number of RMPP cases without the mutation (n=6). We have added a sentence in the **Discussion section (The section highlighted in red in the second paragraph)** and in the **Limitations paragraph (The last two paragraphs of the Discussion section)** to transparently discuss this issue and its implications for the precision of the odds ratio estimate.

**Change made:**

**Results section (3.8):** Added: “Variables with a P-value < 0.05 in the univariate comparisons between the RMPP and non-RMPP groups (as shown in Tables 2, 3, 4, 6, 7) were considered candidates for the multivariate logistic regression model. These included: duration of fever, CRP, LDH, D-dimer, ESR, PCT, pulmonary consolidation, pleural effusion, bronchoscopic alveolar lavage, plastic bronchitis, extrapulmonary complications, and treatment regimen. To avoid overfitting and multicollinearity, we selected the most clinically relevant and statistically robust predictors (macrolide-resistant gene mutation, duration of fever, and D-dimer) for the final model.”

**Section 4 (The second paragraphs of the Discussion section):** Added: “In this study, among patients with RMPP, 71 cases had macrolide-resistant gene mutations while 6 did not, Statistical analysis confirmed a significant association between the presence of macrolide-resistant mutations and RMPP (OR: 17.8, 95% CI: 2.9–107.9). **However, this association requires careful interpretation.** First, while the odds ratio was high (OR: 17.8), its 95% confidence interval was notably wide (2.9–107.9), indicating a degree of imprecision in the estimate, which is likely influenced by the relatively small number of RMPP cases without the mutation (n=6). This underscores the need for validation in larger cohorts. Furthermore, ROC curve analysis for the mutation yielded an AUC of only 0.625 (95% CI: 0.549–0.701), with a sensitivity of 92% but a specificity of 33% (Youden index = 0.250). These results suggest that while the mutation is strongly associated with RMPP, its standalone value for clinical prediction is limited. This limited predictive utility likely reflects the multifactorial pathogenesis of RMPP”.

**Comment 1.4:** “While concluding I feel authors overstate ‘drug-resistant mutation lead to RMPP’ and given the retrospective nature of the study ‘association of mutation’ might sound better.”

**Response:** We agree with the reviewer and appreciate this caution. Our study demonstrates a significant correlation but cannot prove causation. We have toned down the language throughout the manuscript to consistently reflect an “association” rather than a causal relationship.

**Change made:** We have revised relevant statements in the **Abstract, Conclusion, and Discussion**.

**Abstract (Conclusion):** Revised to :“The development of RMPP is **associated with** drug-resistant mutations...”

**Conclusion (Section 5):** Revised to state: “The development of RMPP was independently associated with three key factors: …”

**Discussion:** Checked and revised any inadvertent causal phrasing, emphasizing “association,” “correlation,” and “risk factor.”

**2. Language and Style**

**Comment 2.1:** “Repetition: Some phrases and results are repeated in both the abstract and results section…”

**Response:** We have streamlined the abstract to make it more concise, removing detailed methodological descriptions and focusing on key objectives, findings, and conclusions.

**Change made:** The **Abstract** has been shortened.

**Comment 2.2:** “Tense Consistency: Use past tense for methods and results (‘was conducted,’ ‘showed’) and present tense for interpretations.”

**Response:** We have carefully reviewed the entire manuscript to ensure consistent verb tense usage.

**Change made:** Corrected tense inconsistencies were found and amended, particularly in the **Methods (past tense)** and **Discussion (present tense for general knowledge and interpretation of our results)** sections.

**Comment 2.3:** “Terminology: 1. Replace ‘drug-resistant group’ with ‘macrolide-resistant group’ for precision. 2. Avoid ‘datas’ (plural error in Methods 2.1) → ‘data were accessed.’ 3. Avoid unnecessary capitalization…”

**Response:** Thank you for these precise corrections.

**Change made:**

The term “drug-resistant” has been replaced with “**macrolide-resistant**” throughout the manuscript (e.g., in group names, table titles, and text).

“All datas were accessed…” has been corrected to “All **data** were accessed…” in **Section 2.1**.

We have reviewed and corrected unnecessary capitalization (e.g., “Paediatrics Department” to “department of paediatrics”).

**Comment 2.4:** “Sentence Structure: Some sentences are long and complex. Break them into shorter, clearer statements. Example provided…”

**Response:** We appreciate this suggestion for improving readability. We have revised several long, complex sentences in the manuscript, particularly in the **Introduction, Result, Discussion and Conclusion**, to make them clearer.

**Comment 2.5:** “Abstract: Limit to 300 words. Current abstract slightly exceeds and reads as overly detailed; consider shortening the methods description.”

**Response:** We have significantly shortened the abstract.

**Change made:** The revised **Abstract** is now under 300 words. The methods section was condensed, and redundant phrases were removed.

**3. Data Presentation**

**Comment 3.1:** “Figure and Table Titles: Ensure all tables/figures are self-explanatory and units are standardized (ng/mL, days). Avoid abbreviations in headings (e.g., “PE,” “PB”) unless defined.”

**Response:** We have revised all table and figure titles and footnotes.

**Comment 3.2:** “References: Many references are outdated (some >10 years). Include more recent studies (2022–2024) on macrolide-resistant M. pneumoniae in children, especially from China.”

**Response:** We have updated the reference list to include key recent publications.

**Change made:** We have added several references from 2022-2024 that discuss the epidemiology of macrolide-resistant *M. pneumoniae* and the prediction of RMPP in children, particularly from Chinese research groups

**Comment 3.3:** “Tables are comprehensive but dense. Consider moving some comparisons (e.g., mixed infection data) to supplementary material. ROC figure is appropriate; ensure AUC values are reported with 95% CI.”

**Response:** We agree with these suggestions to improve presentation.

**Change made:** The **95% Confidence Intervals (95% CI) for the AUC values** have been calculated and added to **Table 9** and the corresponding text in **Section 3.9**.

**4. Recommendation**

**Comment 4.1:** “The study addresses an important and under-reported topic with robust data and sound analysis. However, the following are needed before publication: 1. Clarify methodology and statistical modeling. 2. Refine language and streamline redundant sections. 3. Ensure compliance with PLOS ONE data-sharing and formatting guidelines. 4. Tone down causal claims and strengthen limitations discussion.”

**Response:** We are grateful for the reviewer’s overall positive assessment and specific roadmap for revision.

**Change made:** We have addressed all four points as detailed above:

1. **Methodology/Statistics:** Clarified RMPP definition, added molecular assay details, explained variable selection, and discussed wide CIs.
2. **Language/Redundancy:** Improved terminology, tense consistency, sentence structure, and shortened the abstract.
3. **Compliance:** We have formatted the manuscript according to the target journal’s (PLOS ONE) guidelines, including the formatting of references, figures, and the inclusion of required statements (e.g., data availability, competing interests).
4. **Causal Claims/Limitations:** Toned down language to “association,” and **we have added two paragraphs addressing limitations at the end of the discussion section**, significantly expanded the **Limitations** paragraph to explicitly mention the retrospective design, treatment heterogeneity in the non-RMPP group (as previously added per your earlier request), potential selection bias, single-center nature, sample size constraints, and the statistical imprecision indicated by wide confidence intervals.

We believe that through these comprehensive revisions, we have satisfactorily addressed all the concerns raised by the reviewers. Thank you once again for your constructive feedback, which has greatly enhanced our manuscript.
